# Functional State of Lampbrush Chromosomes in Early Vitellogenic Oocytes of Hibernating Frogs *Rana temporaria*

**DOI:** 10.3390/jdb14010007

**Published:** 2026-02-02

**Authors:** Nadya V. Ilicheva, Olga I. Podgornaya

**Affiliations:** Institute of Cytology of the Russian Academy of Sciences, 194064 Saint Petersburg, Russia

**Keywords:** lampbrush chromosomes, amphibians, *Rana temporaria*, transcription, tandem repeats, satellite DNA

## Abstract

Lampbrush chromosomes (LBCs) are a feature of amphibian oocytes and are typically associated with high levels of transcription during active oocyte growth. However, their state during winter hibernation has not been studied. Here, we investigated LBCs in early vitellogenic oocytes (early stage 4) of the grass frog *Rana temporaria* during winter hibernation. We found that the chromosomes retained their lampbrush morphology, and the phosphorylated form of RNA polymerase II resided on the lateral loops. Transcription on the lateral loops was reduced but detectable at cold conditions and significantly increased when the oocytes were transferred at room temperature. Satellite S1a transcripts were detected at the lateral loops of the chromosomes by RNA FISH. The possible significance of maintaining chromosomes in the lampbrush form during hibernation is discussed.

## 1. Introduction

In the diplotene oocytes of many animal species, meiotic bivalents transform into so-called lampbrush chromosomes (LBCs) [1,2]. LBCs are characterized by their large size and distinctive structure. They consist of a central axis composed of highly condensed chromomeres, from which numerous decondensed lateral loops protrude. These lateral loops are sites of active transcription: they are densely packed with RNA polymerase transcribing various types of sequences, which are likely required for future embryo development [1,3]. In addition to ordinary lateral loops, LBCs sometimes contain marker loops, which differ from other loops in the length or thickness of their ribonucleoprotein matrix. Such loops are formed at specific sites on the LBCs and serve as cytological landmarks, enabling the identification and mapping of individual LBCs within a species [1,4,5].

LBCs are characteristic of some invertebrates and all vertebrates except mammals [2], but most research on the structure and function of LBCs has been conducted on avian and amphibian oocytes [1,6]. Due to their large size and unique structure, LBCs have been widely used in studies of chromosome structure and function. For example, LBCs serve as a valuable tool for the high-resolution cytogenetic mapping of chromosomes [4,7], investigation of transcription and post-transcriptional RNA processing [6], and the study of extrachromosomal nuclear bodies [8,9,10].

Although the structure and function of LBCs have been extensively studied, their biological significance is not completely understood. It is known that many non-coding sequences are transcribed on LBCs (reviewed in [2,11]). Most of these sequences are represented by tandem repeats (TRs) and transposable elements (TEs). TRs consist of multiple copies of monomer sequences arranged head-to-tail. Some classes of TRs are historically known as “satellite DNA”; they form heterochromatin blocks in centromeric, pericentromeric, and subtelomeric regions [12]. TE copies are scattered throughout the genome and include DNA transposons and retrotransposons of various classes [13]. Among the repeated sequences transcribed on LBCs are telomeric repeats TTAGGG [5,14,15], PR1, CMN, and PO41 repeats in birds [16,17,18], centromeric and pericentromeric satellites in amphibians [5,19,20], and many others. Some TE transcripts are known to participate in the production of PIWI-interacting RNAs [21,22], but the sense of TR transcripts in oocytes is still unexplored.

It is believed that transcription on LBCs continues for an extended period, much longer than is required to supply the oocyte with transcripts necessary for future embryo development [23]. Early studies have shown that the absolute amount of polyadenylated RNA per oocyte at the onset of vitellogenesis is approximately the same as at the end of oogenesis [24], and that polyadenylated RNA synthesized in the early stages of oocyte development is unusually stable and does not decline for up to 18 months [25]. To explain these phenomena, it may be necessary to expand the range of model organisms or apply new approaches to LBC investigation.

In an attempt to clarify some unresolved issues, we used early vitellogenic oocytes of the grass frog *Rana temporaria*. Unlike commonly used model organisms such as *Xenopus laevis* and *Xenopus tropicalis*, *R. temporaria* is characterized by seasonal breeding and a prolonged period of winter hibernation, making its oocytes particularly suitable for studying LBCs during hibernation. The morphology of *R. temporaria* LBCs has been previously investigated during the period of rapid oocyte growth in the summer, late stage 4 of development according to Duryee [26], and during the final stages of oocyte development (stages 5 and 6) [27]. Stages 5 and 6 occur during hibernation, when LBCs shorten, their lateral loops diminish, and, in the final stage 6, condensed chromosomes form a so-called karyosphere [28,29,30]. In the present study, we examined the state of LBCs in early vitellogenic oocytes (early stage 4) during winter hibernation. All previous studies on LBCs have focused on fast-growing oocytes, when transcriptional activity is high [1,2,6]. While several studies have examined the effect of cold shock on LBC structure and function [31,32,33], no data are available on the structure and function of LBCs in early vitellogenic oocytes during winter hibernation until now. It should be noted that hibernation and experimental cold shock differ significantly. Hibernation is a prolonged gradual process with certain biochemical changes such as cryoprotectant accumulation [34], while cold shock is a stress caused by rapid transfer to the non-physiological conditions. Therefore, the aim of this study was to investigate the structural and transcriptional state of lampbrush chromosomes in early vitellogenic oocytes of *R. temporaria* during winter hibernation. Specifically, we sought to determine whether (i) LBCs retain their structure, (ii) remain transcriptionally active, and (iii) produce TR transcripts, particularly the major pericentromeric satellite S1a, which is the most well-characterized tandem repeat in the *Rana* genus [35,36,37].

## 2. Materials and Methods

### 2.1. Animals

Adult female frogs *R. temporaria* were collected from natural habitats in the Leningrad Region (Russia) in October. Animals were kept in tanks with a small amount of water at +4 °C from October to April. For the experiments with summer oocytes, the frogs were collected from natural habitats in the Leningrad Region in July; the frogs were kept in tanks with a small amount of water at room temperature (RT) and fed with crickets. Information on the number of animals, number of oocytes, and time points of the experiments is given in Table 1.

### 2.2. Isolation of Germinal Vesicles (GVs) and LBC Preparations

Fragments of ovaries were surgically removed and stored in OR2 medium (82.5 mM NaCl, 2.5 mM KCl, 1 mM CaCl_2_, 1 mM MgCl_2_, 1 mM Na_2_HPO_4_, and 5 mM HEPES, pH 7.6). GVs were manually isolated from vitellogenic oocytes at early stage 4 of development according to Duryee’s classification [24]. All procedures were conducted either at room temperature (RT) or on ice, depending on the experiment. The procedure for LBC preparation has been described in detail [38,39]. Briefly, oocytes were placed in a Petri dish with a 5:1 + PO_4_ solution (83 mM KCl, 17 mM NaCl, 6.5 mM Na_2_HPO_4_, 3.5 mM KH_2_PO_4_) with the addition of 1 mM MgCl_2_, and the GVs were isolated from oocytes under a binocular microscope Olympus SZX7 (Olympus, Tokyo, Japain) using a titan needle and forceps. After isolation, each GV was immediately transferred to an isolation chamber containing a 0.25 × 5:1 + PO_4_ solution with 1 mM MgCl_2_ and 0.1% paraformaldehyde (PFA). The nuclear envelope was removed using two titan needles, and the nuclear contents were allowed to disperse for 30 min to 1 h. The chambers were then covered with coverslips and centrifuged at 2000× *g* for 30 min. LBC preparations were fixed with 2% PFA in PBS for 30 min, washed twice for 10 min with PBS, and either mounted with Vectashield containing DAPI (Vector Laboratories, Newark, CA, USA) or processed for immunostaining or FISH. LBCs from at least five GVs were used for each experiment.

### 2.3. Immunofluorescent Staining

After fixation, LBC preparations were blocked in blocking solution (3% BSA, 1× PBS, 0.02% NaN_3_) for 30 min RT and incubated with primary antibodies (Abs) at +4 °C in a moist chamber overnight. The primary Abs were diluted in the same blocking solution. The following primary Abs were used in this study: rabbit monoclonal Abs against the phosphorylated C-terminal domain of RNA polymerase II (Abclonal, Woburn, MA, USA, AP0996), diluted 1:100; and mouse monoclonal anti-bromodeoxyuridine (anti-BrdU) Abs (clone BU-33, Sigma, St. Louis, MO, USA), diluted 1:600. The preparations were washed with PBS (3 × 10 min) and incubated with secondary Abs for 1.5 h at RT. The secondary Abs were goat anti-rabbit or goat anti-mouse IgG conjugated with Alexa Fluor 488 (Abcam, Cambridge, UK). After washing with PBS, the samples were mounted in Vectashield with DAPI.

### 2.4. BrUTP Microinjections

Oocytes were placed in a Petri dish with OR2 medium, and 2.3 nL of 100 mM BrUTP (Sigma) in transcription buffer (140 mM KCl, 2 mM PIPES, pH 7.4) was injected into the oocytes using a Nanoject II microinjector (Drummond, Broomall, PA, USA). Oocytes were then incubated in OR2 medium for either 2.5 h at RT, 2.5 h at +4 °C, or 24 h at +4 °C. After incubation, LBCs were isolated as described above and stained with Abs against BrdU. LBCs isolated from uninjected oocytes stained with primary and secondary Abs were used as a negative control.

### 2.5. Probe Preparation

The probe for the S1a satellite was prepared by PCR using Cy3-labelled dUTP (Lumiprobe, Moscow, Russia) and primers specific to the S1a satellite sequence. Total DNA from *R. temporaria* liver was isolated using the LumiSpin Kit (Lumiprobe, Moscow, Russia) and used as the template for PCR. The primers for S1a were designed in our previous work [37]. The PCR program was as follows: 1 cycle at 95 °C for 5 min; 25 cycles of 95 °C for 30 s, 57 °C for 30 s, and 72 °C for 30 s; followed by 1 cycle at 72 °C for 5 min. The probe was ethanol-precipitated, centrifuged, and diluted in TE buffer (25 mM Tris-HCl, 1 mM EDTA). For preparation of the hybridization mixture, 2 μL of the probe was mixed with 18 μL of Hybrizol (MP Biomedicals, Irvine, CA, USA).

### 2.6. Fluorescent In Situ Hybridization

LBCs were isolated, fixed with 2% PFA in PBS, and washed as described above. After washing, the preparations were dehydrated in a graded ethanol series (50%, 70%, and 95%) for 5 min each. The preparations were then air-dried and processed for FISH. Three variants of the FISH protocol were used: DNA/(DNA + RNA), DNA/RNA, and DNA/DNA [40]. For DNA/(DNA + RNA) FISH, both the LBC preparations and the probe were denatured for 7 min at 80 °C, then hybridized at 37 °C for 18 h in a moist chamber. For DNA/RNA FISH, the hybridization mixture was heated at 95 °C for 10 min, placed on ice for 10 min, and then applied to an undenatured LBC preparation, followed by hybridization at 37 °C for 18 h in a moist chamber. For DNA/DNA FISH, the LBC preparation was incubated with 100 μg/mL RNase A (Sigma-Aldrich, R6513, Merck KGaA, Darmstadt, Germany) for 1 h at 37 °C after PFA fixation, then dehydrated and processed according to the DNA/(DNA + RNA) FISH protocol, as described above. After hybridization, preparations were washed in 2× SSC buffer (0.3 M NaCl, 0.03 M sodium citrate) for 3 × 5 min at 45 °C and mounted in Vectashield with DAPI. As a negative control, LBC preparation was incubated with 100 μg/mL RNase A for 1 h at 37 °C after PFA fixation and then processed through the RNA FISH protocol.

### 2.7. Acquisition and Processing of Images

Preparations were examined using an LSM 5 PASCAL confocal laser-scanning microscope (Carl Zeiss, Oberkochen, Germany) equipped with argon (488 nm) and helium-neon (543 nm) lasers. Images were processed using ImageJ software (v. 1.54g). For BrUTP injection experiments, images of incorporated BrUTP were acquired using identical exposure settings and processed with the same brightness and contrast parameters in ImageJ.

### 2.8. Statistical Analysis

The number of animals used and LBC preparations examined is given in Table 1 (Section 2.1). Statistical analysis of lateral loop lengths and fluorescence intensities was conducted using Excel 2013 software. The statistical difference was calculated by the two-tailed Student’s *t*-test. The levels of significance were set as *p* < 0.05.

## 3. Results

### 3.1. Structure of LBCs in the Oocytes of Hibernating Frogs R. temporaria

During winter hibernation, the ovary of *R. temporaria* contains immature oocytes (stages 1–4 of oocyte development according to Duryee [26] and fully grown oocytes at stage 5 or stage 6, which will ovulate in spring. We selected early stage 4 oocytes, which are 0.5–0.6 mm in diameter and have a light grey color due to the onset of yolk accumulation. Because oocytes do not grow significantly during winter hibernation, we aimed to determine whether meiotic chromosomes during this period maintain the lampbrush form, which is typically associated with active transcription.

**Figure 1:**
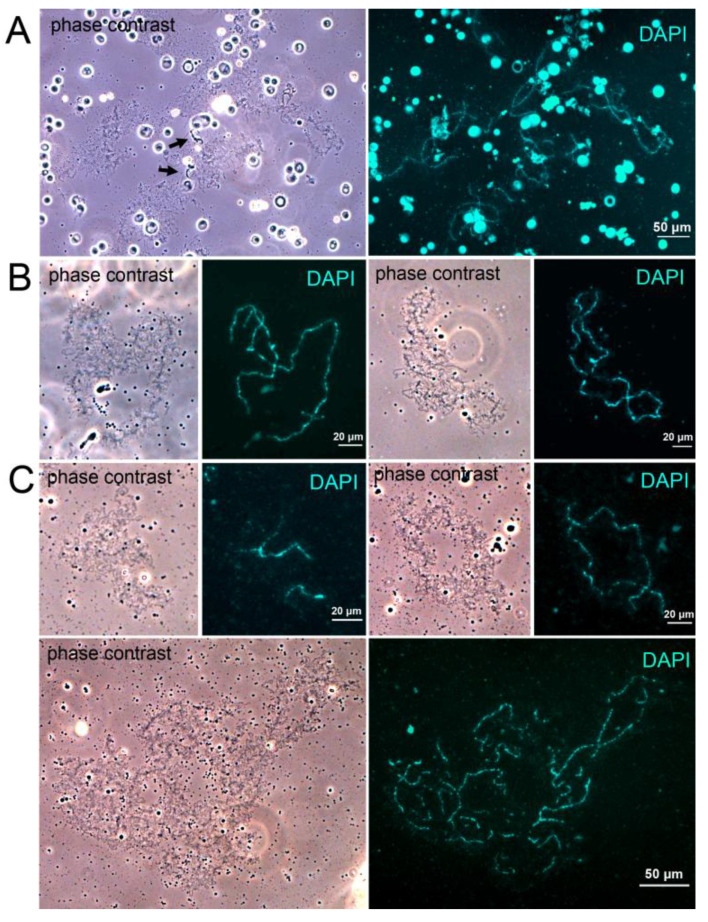
Lampbrush chromosomes (LBCs) in *Rana temporaria* oocytes. (**A**) LBCs isolated from stage 4 oocytes in the period of fast growth in summer. The arrows point to marker loops on one of the LBCs. (**B**,**C**) LBCs isolated from stage 4 oocytes during winter hibernation. LBCs were isolated either at room temperature (**B**) or on ice (**C**).

We used the classical LBC isolation protocol developed by Callan and co-authors [38] for amphibian oocytes. We found that the isolated meiotic chromosomes retained their lampbrush form, with prominent lateral loops (Figure 1). However, their lateral loops were shorter than the lateral loops of LBCs isolated in summer: average lateral loop length was 19.9 μm for winter oocytes and 35.4 μm for summer oocytes (Supplementary Figure S1). Also, we did not observe prominent marker loops, particularly lumpy loops, intrinsic for LBCs isolated from summer stage 4 oocytes (Figure 1A, Supplementary Figures S2 and S3). In our initial experiments, LBCs were isolated at room temperature (RT) (Figure 1B). To confirm that the presence of lateral loops was not an artifact caused by warming of the oocytes, we next isolated LBCs on ice. Under cold conditions, the nuclear content dispersed more slowly, and chromosomes were often entangled, but they still retained the lampbrush form (Figure 1C).

### 3.2. Localization of the Active Form of RNA-Polymerase II

**Figure 2:**
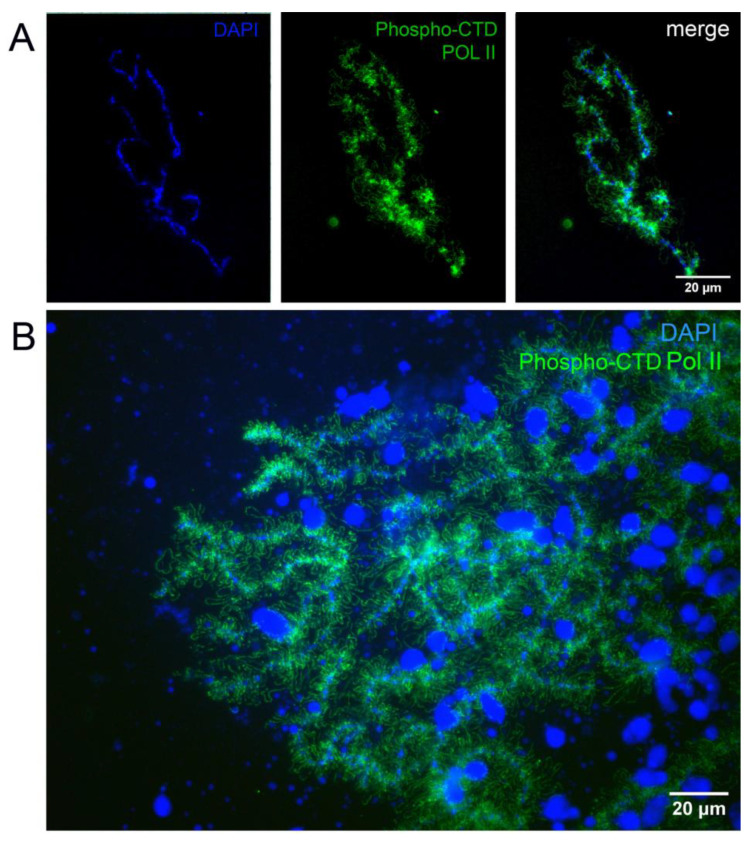
Distribution of the active form of RNA polymerase II (phosphorylated at the C-terminal domain) on the LBCs of hibernating *R. temporaria*. The LBCs were isolated at room temperature (**A**) and on ice (**B**).

We used antibodies against the phosphorylated C-terminal domain (CTD) of RNA polymerase II (Pol II) to detect the active form of Pol II on the LBCs of hibernating *R. temporaria* oocytes. As shown in Figure 2, the antibodies stained the lateral loops of LBCs in oocytes isolated both at RT and on ice. Staining was observed in all examined LBC preparations. Therefore, we conclude that Pol II located on the lateral loops retains its phosphorylated state, which is characteristic of the actively elongating form.

### 3.3. Transcription on the LBCs

**Figure 3:**
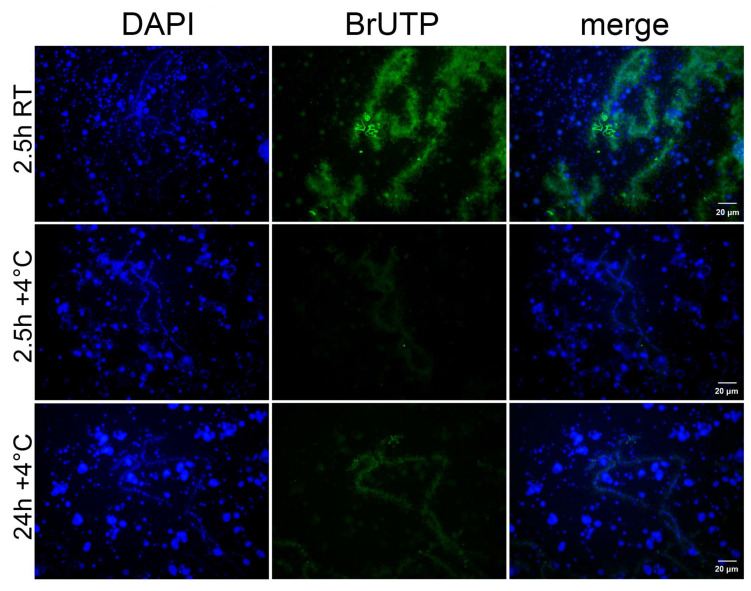
Transcription on the LBCs of hibernating *R. temporaria*. The first panel (2.5 h RT) represents BrUTP incorporation in the lateral loops of LBCs from injected oocytes incubated for 2.5 h at room temperature. The second panel represents BrUTP incorporation in the lateral loops of LBCs from injected oocytes incubated for 2.5 h at +4 °C. The third panel represents BrUTP incorporation in the lateral loops of LBCs from injected oocytes incubated for 24 h at +4 °C.

To assess transcriptional activity on LBCs, we performed BrUTP microinjections into the ooplasm. After 2.5 h of incubation at RT, significant incorporation of BrUTP into the lateral loops of the LBCs was observed, with some loops showing more intense staining than others (Figure 3). When injections were performed on ice and oocytes were incubated at +4 °C for 2.5 h, no significant BrUTP incorporation was detected. However, 2.5 h may be insufficient to detect BrUTP incorporation at +4 °C, as Pol II activity is likely reduced at low temperatures [41]. Therefore, we extended the incubation time to 24 h at +4 °C. Under these conditions, BrUTP incorporation was observed, although the signal was notably weaker than in oocytes incubated for 2.5 h at RT. The results were confirmed by quantitative assessment of fluorescence intensity (Supplementary Figures S4 and S5). We conclude that *R. temporaria* LBCs retain transcriptional activity even under cold conditions during winter hibernation.

### 3.4. Transcription of S1a Satellite

**Figure 4:**
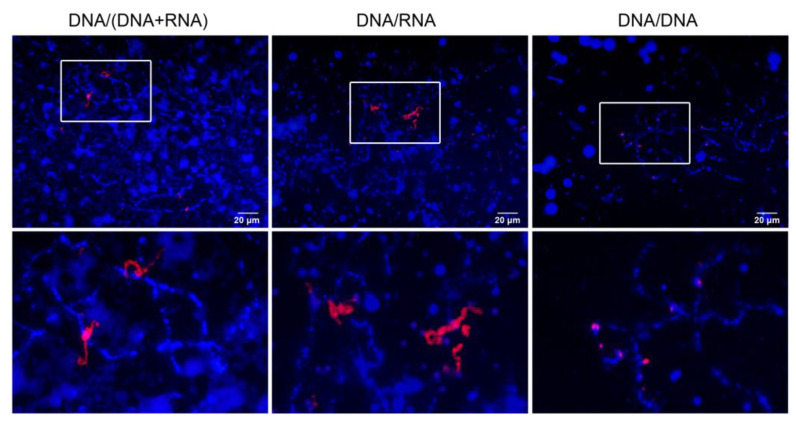
FISH detection of S1a satellite transcripts on the LBCs of hibernating *R. temporaria* oocytes. FISH was performed according to the protocols of DNA/(DNA + RNA), DNA/RNA, and DNA/DNA hybridization (control). The lower panel represents enlarged areas highlighted by white boxes.

Transcription of tandem repeats on LBCs has been demonstrated in many species [2], and we aimed to determine whether sequences of this type are transcribed in *R. temporaria* oocytes during hibernation. In this study, we used a probe specific to the S1a satellite. To detect S1a transcripts on the LBCs, we applied DNA/RNA and DNA/(DNA + RNA) FISH protocols, while DNA/DNA FISH was performed as a control (Figure 4). We found that the S1a satellite is transcribed in all early vitellogenic oocytes examined by DNA/RNA and DNA/(DNA + RNA) FISH. In preparations pretreated with RNase, the probe stained chromomeres composed of S1a arrays. Negative control (hybridization of a denatured probe with RNase-treated undenatured LBCs) gave no staining.

## 4. Discussion

The chromosomes of *R. temporaria* oocytes retain their lampbrush form during winter hibernation, a period characterized by reduced metabolic activity. Although several studies have examined the effects of cold conditions on oocytes, these have primarily focused on cold shock in animals not in a state of hibernation. In [31,33], females of the newt *Pleurodeles waltl* were exposed to low temperatures (+8 °C) for several days. The authors observed a reduction in both the size and number of normal lateral loops, along with the formation of large “cold loops” at specific chromosomal loci. It is important to note that newt LBCs exhibit various loop types with distinct morphologies, such as granular and globular loops, which are often used as markers for identifying individual LBCs [4]. Cold loops were shown to arise specifically from granular loops [31]; thus, in species lacking this loop type, cold loops may not form at all. In a separate study, Liu and Gall [32] investigated the effect of cold shock on isolated *X. laevis* oocytes. Oocytes were placed at +4 °C for relatively short periods (12 h to 1 day). While no major changes in LBC morphology were observed, the authors reported the formation of unique structures, cold bodies (C-bodies), at specific sites on the LBCs. In our study, we did not observe any structures resembling cold loops or C-bodies on the LBCs of hibernating *R. temporaria*. However, the structure of LBCs during hibernation differed from that of actively growing oocytes. Firstly, the lateral loops of LBCs isolated during hibernation were significantly shorter than lateral loops of summer stage 4 oocytes. This may be associated with a decrease in metabolic activity during hibernation. We also did not detect the marker loops typically seen in LBCs from summer stage 4 oocytes (Figure 1A). It is not clear why the LBCs of hibernating frogs miss these structures. Marker loops are known to be enriched with the proteins participating in RNA metabolism, such as hnRNPs [17,42] and CELF1 [43]. The absence of the marker loops may be caused by a low transcription rate during hibernation, so RNPs required for transcript processing are not necessary in huge amounts. Another explanation is that marker loops may depend on the stage of oocyte development and form later in the fast-growing oocytes during summer.

DNA sequences on the lateral loops of LBCs are actively transcribed [1,6]. The majority of these sequences are transcribed by RNA polymerase II, while a small part is transcribed by RNA polymerase III [44,45]. The largest subunit of Pol II has a C-terminal domain, which becomes phosphorylated in the elongating form of the polymerase. In this study, we found that the active form of Pol II was present on the lateral loops of LBCs in hibernating frogs. Experiments on BrUTP microinjections with following incubation of injected oocytes at +4 °C during 24 h revealed a weak labeling on the lateral loops. We also observed that when oocytes were incubated at RT, active transcription on the lateral loops resumed. Some loops showed more intense staining than others (Figure 3), which may indicate higher transcriptional activity at those sites. This suggests that the transcriptional machinery remains in place on the lateral loops, poised to function when conditions become favorable. In a related study on cold body formation in *X. laevis* oocytes, Liu and Gall [32] performed BrUTP injections into cold-shocked oocytes. After one day of incubation at low temperature, they observed a bright BrUTP signal at the lateral loops, comparable to that of control oocytes incubated at RT. In contrast, our study showed significantly weaker BrUTP labeling after 24 h of incubation at +4 °C compared to oocytes incubated at RT. Therefore, our results cannot be explained by a simple decrease in Pol II transcription rate at low temperature. This difference in the results could reflect the difference between hibernation and cold shock. Hibernation continued for several months in our experiments, while cold shock lasted for quite a short time. Consequently, changes that lead to a significant reduction in transcription rate may not yet have occurred in the cold-shocked oocytes.

It is not entirely clear why the chromosomes of stage 4 oocytes maintain a transcriptionally active state during winter hibernation. One possible explanation is that a low level of transcription is required to sustain RNA turnover in the oocyte. However, it is known that transcripts produced during the early stages of oocyte development are unusually stable [25]. In our previous works [28,46], we investigated transcription in late vitellogenic oocytes during winter hibernation. During this period (stages 5 and 6 of oocyte development), chromosomes become condensed and form the karyosphere. At stage 5, transcriptional activity is significantly reduced, and by stage 6, chromosomes are transcriptionally silent, although rRNA synthesis in the amplified nucleoli continues even at this stage. This indicates that transcription on the chromosomes is not required to maintain RNA turnover in fully grown oocytes at the final stages of development. Therefore, one might expect transcription to decrease and the transcriptional machinery to be removed from stage 4 oocytes during hibernation. Nevertheless, chromosomes persist in the lampbrush form, with only minimal residual transcription detectable (Figure 3), suggesting that the lampbrush state is not maintained solely for transcript accumulation. Thus, it is possible that the maintenance of lampbrush chromosomes in hibernating oocytes serves a function other than maternal RNA production.

It is well-established that many repeated DNA sequences, particularly TRs, are transcribed on LBCs [2,11]. Satellite S1a is the only cloned TR known in the genus *Rana* [47] and was recently described as TR 494A [37]. In this study, we detected transcripts of this TR on the LBCs of early vitellogenic *R. temporaria* oocytes. The functional significance of widespread TR transcription in oocytes remains unclear. One explanation is offered by the “read-through” hypothesis, which suggests that transcription does not terminate precisely at the 3′ end of genes but instead continues into downstream, non-coding DNA regions [1,48]. This hypothesis assumes that only protein-coding genes have defined transcriptional start sites, though this is not universally accepted [49]. Interestingly, TRs possess features similar to scaffold/matrix attachment regions (S/MARs), and together they may contribute to organizing the 3D nuclear architecture [50]. Another hypothesis proposes that TR transcripts are required for the formation of heterochromatin in the developing embryo [18]. In support of this, it has been shown that in 2-cell mouse embryos, major satellite sequences are actively transcribed, and their transcripts are essential for chromocenter formation and proper embryonic development [51]. In amphibians, zygotic genome activation occurs relatively late, during the mid-blastula transition. Therefore, all factors necessary for early development should be synthesized in the oocyte in large quantities in advance. In addition to their role in embryo development, TR transcripts could participate in subsequent stages of oocyte development. For example, these transcripts may be involved in chromatin compactization during karyosphere formation.

## 5. Conclusions

Despite more than a century of extensive research, many questions regarding the role of LBCs in oogenesis remain unanswered. The most widely accepted classical hypothesis posits that extensive transcription on LBCs is required to supply the oocyte with mRNAs and regulatory RNAs necessary for future embryonic development [3,6,52]. An alternative, the epigenetic hypothesis, suggests that the transformation of oocyte chromosomes into the lampbrush form is essential for the epigenetic reprogramming of the maternal genome [2,53]. According to this view, transcription on LBCs serves to maintain chromatin in an extended state, ensuring access of the nucleoplasm to DNA, while the massive ribonucleoprotein (RNP) complexes help prevent the collapse of the lateral loops. There is some experimental evidence in favor of this hypothesis. It was shown that chromatin of various cell types is reprogrammed when injected into the amphibian oocyte [54,55,56]. Our findings are consistent with this hypothesis, suggesting that the lampbrush form of chromosomes is maintained not only for transcript production but also to preserve an open chromatin state during hibernation. While this explanation does not preclude additional functions such as RNA accumulation for embryo development, it emphasizes that the significance of LBCs may extend beyond bulk RNA synthesis.

## Figures and Tables

**Table 1:** Number of animals and GVs used in the study.

Experiment	Number of Frogs	Number of GVs	Month
LBC structure	2 (summer)	25	July
	3 (hibernating)	10 (isolated at +4 °C) 10 (isolated at RT)	January, February, March
Pol II staining	3 (hibernating)	10 (isolated at +4 °C) 10 (isolated at RT) 5 (control)	January, March
BrUTP incorporation	3 (hibernating)	15 (2.5 h TR) 10 (2.5 h +4 °C) 10 (24 h +4 °C) 5 (control)	March
FISH	3 (hibernating)	20 (DNA + RNA) 10 (RNA FISH) 5 (DNA FISH) 5 (control)	March, April

## Data Availability

Data are contained within the article or Supplementary Materials.

## Supplementary Materials

The following supporting information can be downloaded at: https://www.mdpi.com/article/10.3390/jdb14010007/s1. Figure S1. Lengths of the LBC lateral loops from winter (hibernating) and summer oocytes. 50 random LBC lateral loops from at least three preparations were measured in each category. Loops from hibernating oocytes were measured on the preparations isolated at +4 °C. The distances were measured using ImageJ software; *p* < 0.0001. Figure S2. Full set of Rana temporaria LBCs from summer oocytes. A. Phase contrast. Lumpy loops are shown by arrows. B. DAPI staining. Scale bar: 50 μm. Figure S3. Full set of LBCs from hibernating Rana temporaria oocyte. A. Phase contrast. B. DAPI staining. Figure S4. Quantitative measurements of fluorescence intensity in the experiments on BrUTP incorporation. Fluorescence intensity was measured on raw images using ImageJ software. Plots representing distribution of fluorescence intensity signal are presented on the right. Figure S5. Mean fluorescence intensity of BrUTP incorporation in the oocytes incubated 2.5 h RT, 2.5 h at +4 °C and 24 h at +4 °C after injection. 26 LBCs from two different experiments were used for measurement in each category. LBC preparations from uninjected oocytes stained with primary and secondary Abs were used as a control. Mean fluorescence intensity was measured using ImageJ software. Mean fluorescence intensity in the oocytes incubated 2.5 h RT significantly differs from that of oocytes incubated 2.5 h at +4 °C (*p* < 0.0001) and 24 h at +4 °C (*p* < 0.0001); fluorescence intensity between oocytes incubated 2.5 h at +4 °C and 24h at +4 °C also significantly differ (*p* < 0.0001). There was no significant difference in staining between oocytes incubated 2.5 h at +4 °C and control (*p* = 0.2).

## Abbreviations

The following abbreviations are used in this manuscript:

LBCs	Lampbrush chromosomes
TR	Tandem repeat
TE	Transposable element
GV	Germinal vesicle
RT	Room temperature
Abs	Antibodies
FISH	Fluorescent in situ hybridization
CTD	C-terminal domain
Pol II	RNA polymerase II
S/MAR	Scaffold/matrix attachment region
