# Supplementary material for: Functional State of Lampbrush Chromosomes in Early Vitellogenic Oocytes of Hibernating Frogs Rana temporaria
Source: J Dev Biol. 2026 Feb 2;14(1):7. doi: 10.3390/jdb14010007 (PMC12921996; doi:10.3390/jdb14010007)
Supplement: Supplementary file 1 [file jdb-14-00007-s001.zip › jdb-4016439-supplementary.pdf]

Supplementary material

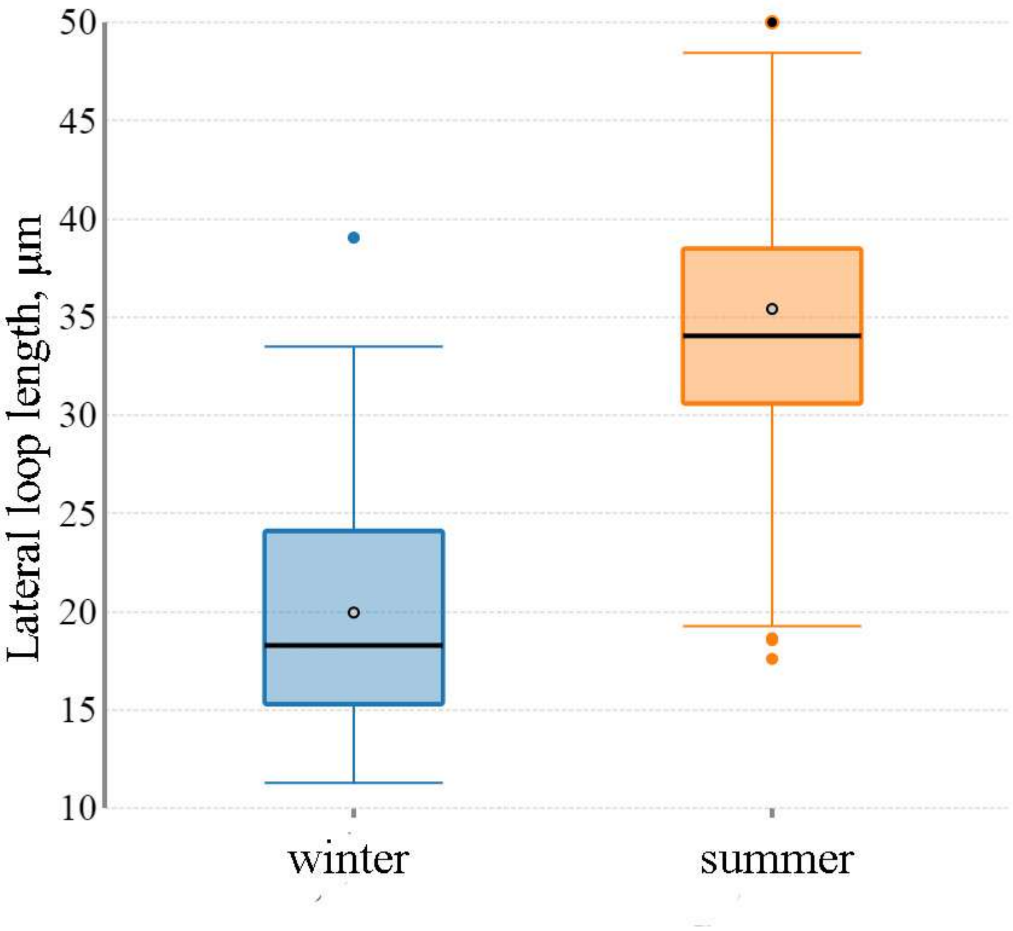

Figure S1. Lengths of the LBC lateral loops from winter (hibernating) and summer oocytes. 50 random LBC lateral loops from at least three preparations were measured in each category. Loops from hibernating oocytes were measured on the preparations isolated at +4°C. The distances were measured using ImageJ software; $p < 0.0001$.

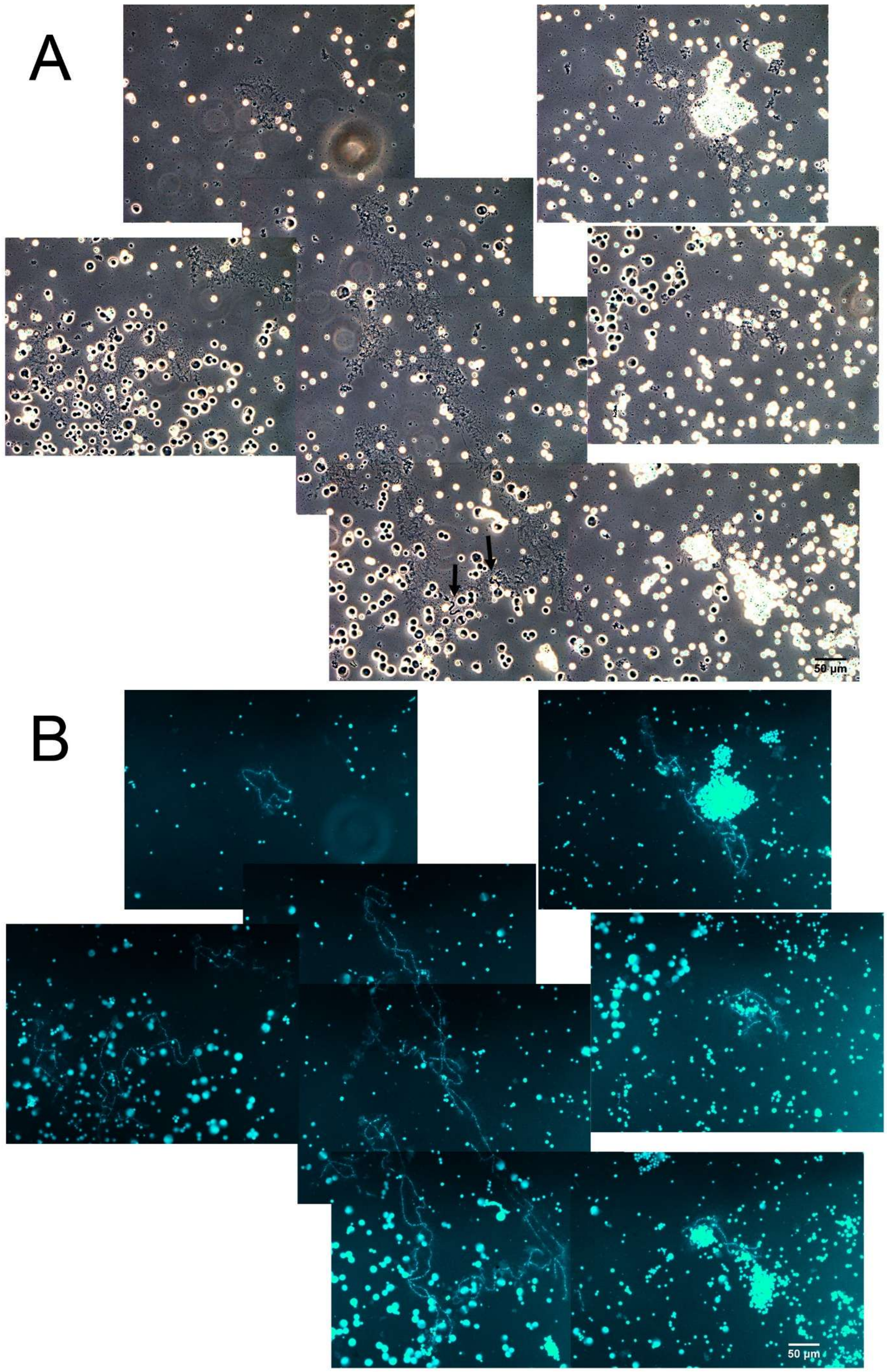

Figure S2. Full set of *Rana temporaria* LBCs from summer oocytes. A. Phase contrast. Lumpy loops are shown by arrows. B. DAPI staining. Scale bar: 50 µm.

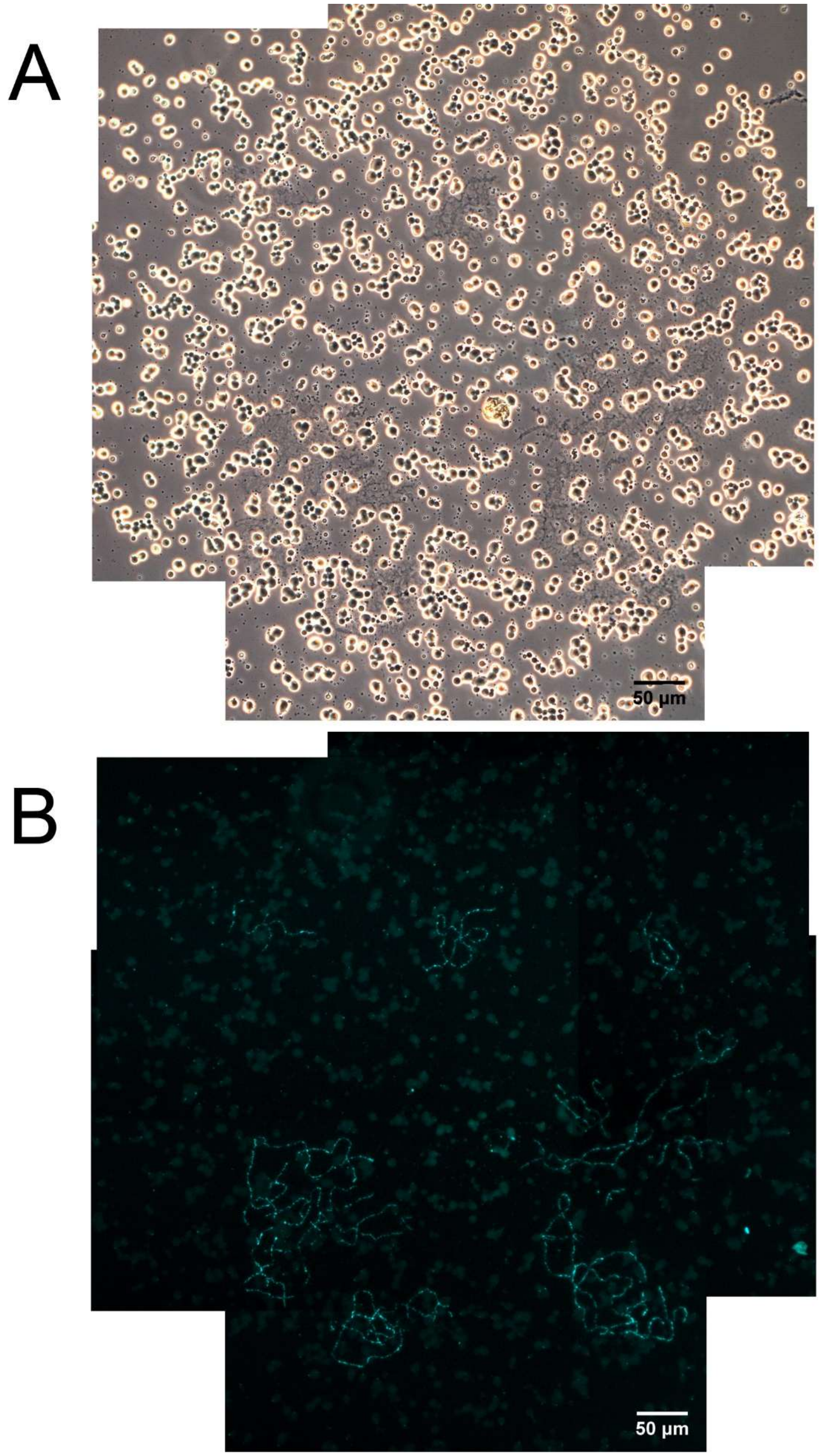

Figure S3. Full set of LBCs from hibernating *Rana temporaria* oocyte. A. Phase contrast. B. DAPI staining.

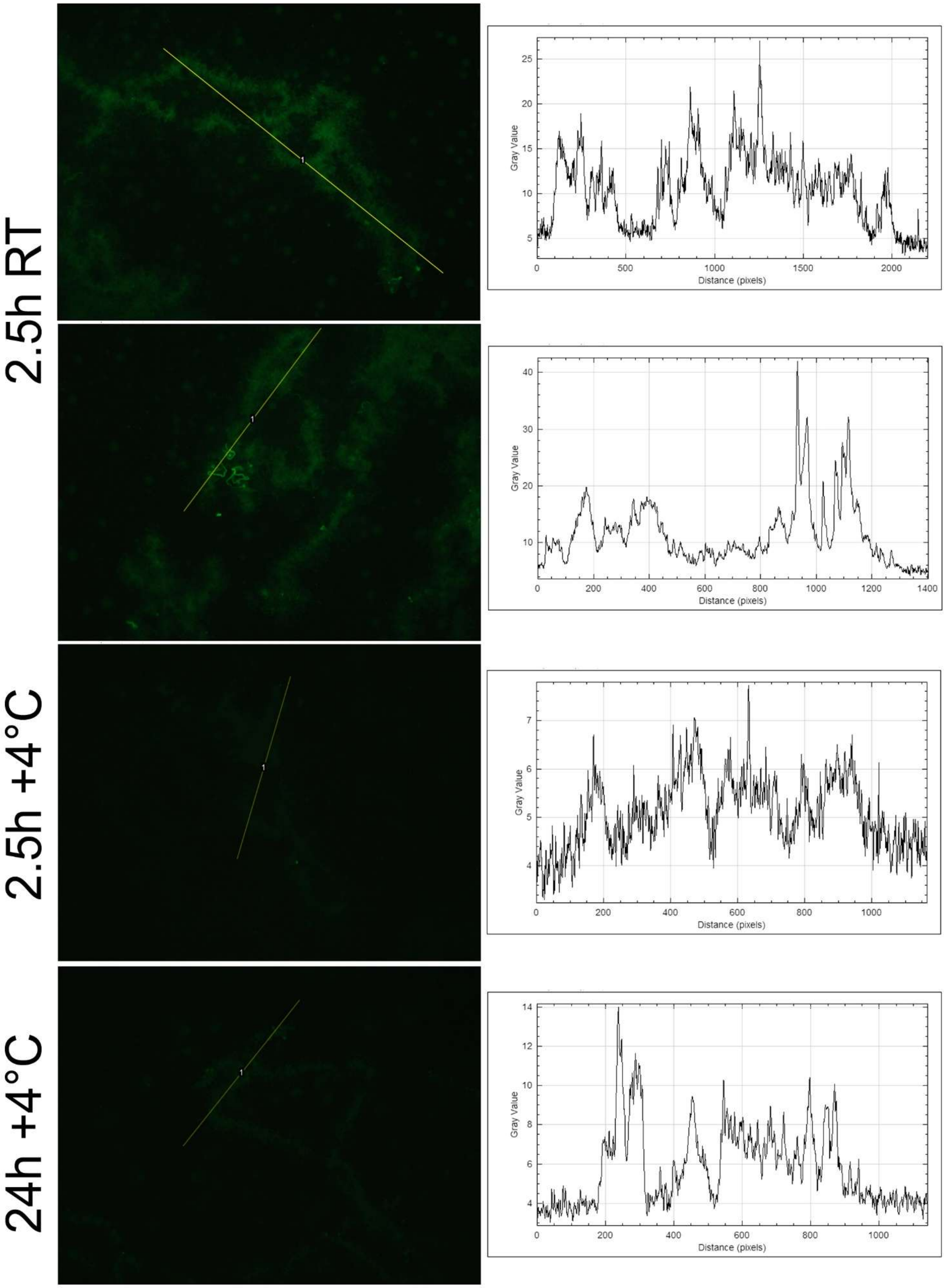

Figure S4. Quantitative measurements of fluorescence intensity in the experiments on BrUTP incorporation. Fluorescence intensity was measured on raw images using ImageJ software. Plots representing distribution of fluorescence intensity signal are presented on the right.

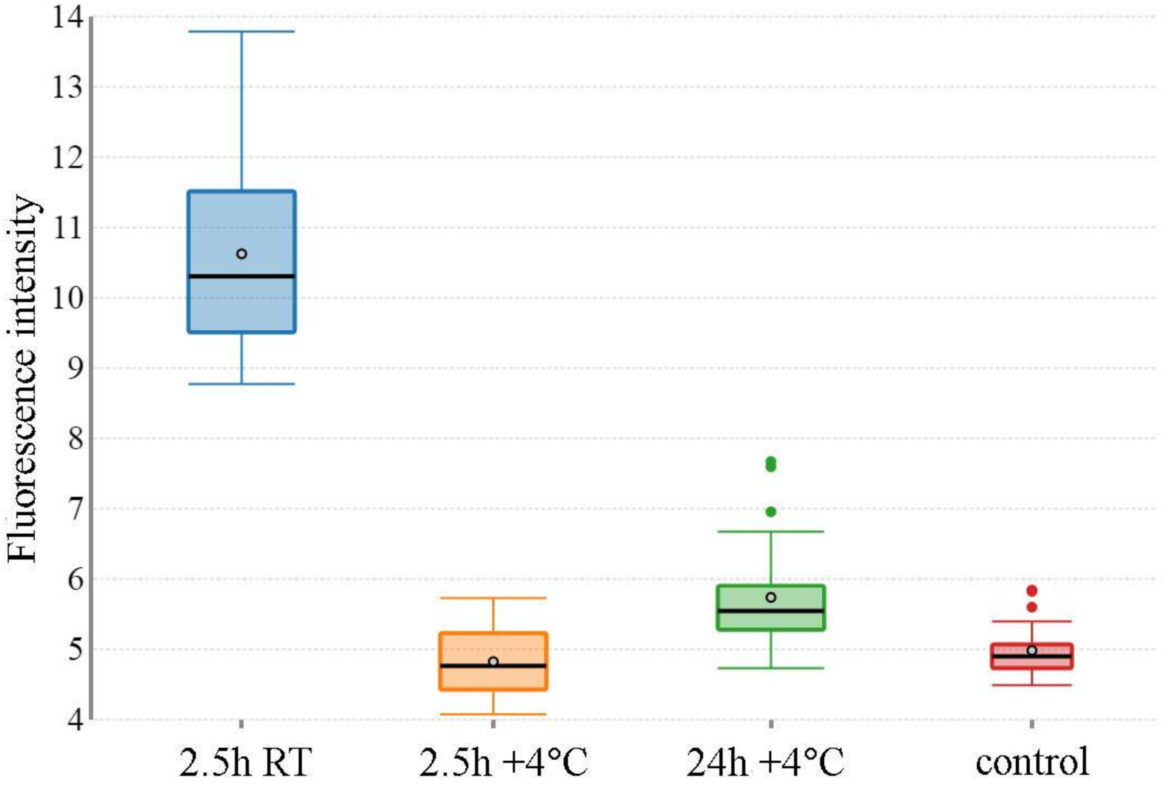

Figure S5. Mean fluorescence intensity of BrUTP incorporation in the oocytes incubated 2.5h RT, 2.5h at +4°C and 24h at +4°C after injection. 26 LBCs from two different experiments were used for measurement in each category. LBC preparations from uninjected oocytes stained with primary and secondary Abs were used as a control. Mean fluorescence intensity was measured using ImageJ software. Mean fluorescence intensity in the oocytes incubated 2.5h RT significantly differs from that of oocytes incubated 2.5h at +4°C ($p < 0.0001$) and 24h at +4°C ($p < 0.0001$); fluorescence intensity between oocytes incubated 2.5h at +4°C and 24h at +4°C also significantly differ ($p < 0.0001$). There was no significant difference in staining between oocytes incubated 2.5h at +4°C and control ($p = 0.2$).
